# Both orf-I isoforms, p12 and p8, are required for efficient HTLV-1 infection and persistence

**DOI:** 10.1186/1742-4690-11-S1-O13

**Published:** 2014-01-07

**Authors:** Cynthia Pise-Masison, Maria Fernanda de Castro-Amarante, Cody Buchmann, Yoshimi Enoso-Akahata, Claudio Fenezia, Dustin Edwards, Luiz Carlos Junior Alcantara, Izabela Bialuk, Steven Jacobson, Bernardo Galvao-Castro, Antoine Gessain, Genoveffa Franchini

**Affiliations:** 1 Animal Models and Retroviral Vaccines Section, National Cancer Institute, Bethesda, MD, USA; 2 Oswaldo Cruz Foundation Salvador, Bahia, Brazil; 3 HTLV Center/ Bahia School of Medicine and Public Health, Salvador, Bahia, Brazil; 4 Department of General and Experimental Pathology, Medical University of Bialystok, Bialystok, Poland; 5 Viral Immunology Section, Neuroimmunology Branch, National Institute of Neurological Disorders and Stroke, Bethesda, MD, USA; 6 Epidémiologie et Physiopathologie des Virus Oncogènes, CNRS URA3015, Pasteur Institute, Paris, Cedex 15, France

## 

HTLV-1 orf-I is important for immune evasion, viral replication and persistence. Here, we analyzed proviral sequences isolated from ex vivo PBMC samples of 163 HTLV-1-infected patients (85 carriers, 78 HAM/TSP). Of the 5000 sequences analyzed, orf-I expression was consistently maintained. While the highest percentage of isolates express both p12 and p8 (69%), the mutants that express mainly p8 (7%) or mainly p12 (24%) were highly correlated with decreased proviral loads. To determine the individual role of p12 and p8 in viral persistence, we constructed infectious molecular clones expressing either p12(G29S) or p8(N26) and established producer cell lines used to infect macaques. Three of four macaques infected with WT-HTLV-1 tested positive by PCR and were seropositive. Only one of the macaques infected with N26-HTLV-1 tested positive by PCR and two macaques were seropositive. None of the 8 animals infected with G29S-HTLV-1 tested PCR positive or seroconverted. These results suggest that expression of both p12 and p8 are critical for viral persistence. Since orf-I plays a role in T-cell activation and recognition, we compared the CTL responses elicited by CD4^+^ T-cell infected with the different HTLV-1 clones. Although the supernatant p19 levels and proviral loads for all four infected lines were similar, a significant difference in Tax-specific CTL killing was observed. Cells infected with orf-I-knockout virus, p12-G29S or p8-N26 were killed whereas cells infected with WT virus were not killed by Tax-specific CTLs. These results indicate that efficient viral persistence and spread requires expression of both p12 and p8.

